# TNF Pathway‐Mediated Tolerogenic T‐Cell Trajectory Driven by Allergen Immunotherapy

**DOI:** 10.1111/all.70367

**Published:** 2026-04-30

**Authors:** Helen S. Charles, Amr A. Gabr, Shu‐Hung Wang, Ulrich M. Zissler, Sonja Heine, Alexander Heldner, Sebastian Kotz, Lisa Pechtold, Lynn S. zur Bonsen, Dimitrii Pogorelov, Josephine Kau, Mirjam Plaschke, Miriam Hills, Ferdinand Guerth, Madlen Oelsner, Caspar Ohnmacht, Francesca Alessandrini, Simon Blank, Adam M. Chaker, Carsten B. Schmidt‐Weber, Constanze A. Jakwerth

**Affiliations:** ^1^ Center of Allergy and Environment (ZAUM), School of Medicine and Health, Technical University of Munich and Helmholtz Munich Munich Germany; ^2^ German Center for Lung Research (DZL) Giessen Germany; ^3^ Department of Physiology Faculty of Veterinary Medicine, Cairo University Giza Egypt; ^4^ Department of Otorhinolaryngology TUM School of Medicine and Health, Klinikum Rechts der Isar, Technical University of Munich Munich Germany; ^5^ Department of Dermatology and Allergy Biederstein School of Medicine, Technical University of Munich Munich Germany

**Keywords:** allergen immunotherapy, exhaustion, T cells, Th17 cells, trans‐differentiation

## Abstract

**Background:**

Allergen immunotherapy (AIT) is a therapeutic approach to restore allergen tolerance and prevent asthma progression. Previous studies have shown exhaustion of T cells and the induction of T cells expressing IL‐17 and FOXP3 early in AIT, which are relevant for the clinical outcome. This study aims to investigate the dynamic transition from type‐3 immunity to a regulatory state observed in the first year during allergic inflammation, as well as the subsequent dysfunction of effector cells during AIT.

**Methods:**

Human and experimental models of allergic airway inflammation were used to assess the impact of AIT on Treg, Tr17 and Th17 cell populations using flow cytometry and proliferation assays. Additionally, human blood samples were analysed using single‐cell transcriptomics to characterise transcriptional signatures associated with the transition from pro‐inflammatory to regulatory states.

**Results:**

AIT restored balance of Tr17 and Treg populations and increased their proliferative capacity, whereas Th17 cells remained functionally impaired. Single‐cell transcriptomics identified Tr17 cells as intermediate states between pro‐inflammatory and regulatory T‐cell programs after AIT. In parallel, AIT reprogrammed intracellular communication networks, with TNF/LTA‐associated signalling pathways emerging as prominent mediators of tolerogenic signalling.

**Conclusion:**

These findings highlight that AIT reprograms immune responses by enhancing regulatory dominance, inducing Tr17 plasticity and leveraging TNF/TNFR2‐mediated tolerance. Understanding the cellular dynamics during AIT suggests that therapeutic strategies aimed at targeting Th17 functional impairment could further enhance treatment efficacy for allergic airway diseases. This insight opens new avenues for refining immunotherapeutic approaches to more effectively restore immune balance and improve patient outcomes.

## Graphical abstract

The study shows that AIT re‐establishes local immune equilibrium by restoring Th17/Treg balance, while circulating Th17 cells remain functionally impaired. The transition from pro‐inflammatory Th17 to regulatory T cell states during AIT identifies Tr17 cells as an intermediate cell population. AIT reorganises LT‐α‐associated TNFR2/HVEM signalling dynamics, contributing to the establishment of immune tolerance. Abbreviations: AAI, allergic airway inflammation; AIT, allergen immunotherapy; BAL, bronchoalveolar lavage; CTLA‐4, cytotoxic T‐lymphocyte associated protein 4; LT, lymphotoxin; mRNA scSeq: mRNA, single‐cell sequencing; NA, non‐allergic; OVA, ovalbumin; PBMCs, peripheral blood mononuclear cells; PD‐1, programmed death‐1; Treg, regulatory T cell; Th17, T helper 17; TNF, tumour necrosis factor; Tr17, transitional FOXP3 ^+^ IL17A ^+^ 17 cells.

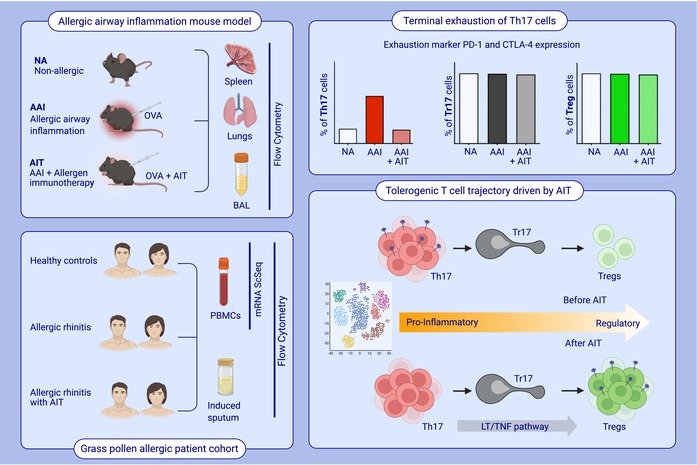

**Abbreviations**
AA
: Allergic asthma
AAI
: Allergic airway inflammation
AIT
: Allergen Immunotherapy
AR
: Allergic rhinitis
BAL
: Bronchoalveolar Lavage
CCR
: C‐C Chemokine Receptor
CTL
: Cytotoxic T Lymphocyte
CTLA‐4
: Cytotoxic T‐Lymphocyte Associated Protein 4
FOXP3
: Forkhead Box P3
HVEM
: Herpesvirus Entry Mediator
iTregs
: Induced Regulatory T cells
LAG‐3
: Lymphocyte‐Activation Gene‐3
LT
: Lymphotoxin
PD‐1
: Programmed Death‐1
T2‐type asthma
: Type‐2 Asthma
Th17
: T Helper 17
Th2
: T Helper 2
TIM‐3
: T‐cell Immunoglobulin and Mucin‐Domain Containing Protein 3
TNF
: Tumour Necrosis Factor
TNFRSF14
: Tumour Necrosis Factor Receptor Superfamily Member 14
TNFRSF1B
: Tumour Necrosis Factor Receptor Superfamily Member 1B
Tr17
: Transitional FOXP3+ IL17A+ 17 Cells
Treg(s)
: Regulatory T Cells

## 1 Introduction

Allergen immunotherapy (AIT) is an established tolerogenic treatment for allergic airway diseases and allergic rhinitis (AR), inducing long‐term immune tolerance to specific allergens [1, 2]. AIT shifts the immune response from a pro‐inflammatory Th2 profile toward a more regulatory phenotype. Recent single‐cell transcriptomic studies [3, 4] have provided insights into these cellular and molecular changes, which complement clinical observations of reduced symptoms and complications. This regulatory phenotype replaces the type‐2 immune response, which creates a pro‐inflammatory environment through IL‐4‐driven IgE, activation of mast cells and infiltration of eosinophils [5]. However, recent research indicates that other T‐cell subsets such as T helper 17 (Th17) cells and regulatory T‐cells (Tregs), significantly contribute to allergic airway disease pathogenesis [6, 7].

Type‐3 innate immune responses are central to the initiation and propagation of inflammatory cascades in autoimmunity, ultimately contributing to tissue damage when regulation fails [8]. In effect, the innate immune reaction in many autoimmune disorders is largely reflected by these type‐3 immune responses, which help to sustain chronic inflammatory environments [9]. In airway allergic diseases, such as neutrophilic asthma, Th17 cells contribute to airway inflammation and neutrophilic responses [10]. On the contrary, Tregs are essential in counteracting the pro‐inflammatory effects driven by the effector Th2 and Th17 cells [11]. We and others previously identified a ‘Tr17’ population co‐expressing *IL‐17A* and *FOXP3* that represents a transitional phenotype between Th17 and Treg cells [12, 13]. These cells emerge during the first year of AIT and have been associated with early prediction of treatment success, suggesting a potential role in restoring immune tolerance.

Chronic antigen exposure can induce dysfunctional or hyporesponsive T‐cell states characterised by altered cytokine production, reduced proliferation and expression of inhibitory receptors such as PD‐1 and CTLA‐4 [14, 15]. Understanding the interactions between these various T cell subsets and their state of exhaustion during the course of AIT could potentially lead to optimisation of treatment efficiency for allergic diseases.

Building on our previous research, peripheral frequencies of Th17 and Tr17 cells in patients who received AIT correlated with the clinical success of the treatment defined by the *Retrospective Assessment of seasonal Allergic Symptoms* (RAAS) score [13]. Our current study investigates the role of these subsets at the site of allergic inflammation and their state of exhaustion during the course of immunotherapy. Our murine models coupled with human sputum and blood analyses reveal that allergic inflammation drives strong local recruitment of these subsets, while the spleen shows higher Treg frequencies along with signs of Th17 dysfunction. AIT restores the cellular balance and proliferative capacities of Tr17 and Treg cells; however, the Th17 subset remains functionally impaired. Single‐cell transcriptomics are consistent with an intermediate transitional state of Tr17 cells capable of shifting between pro‐inflammatory Th17 and regulatory Treg states following AIT. This plasticity potentially plays a prominent role in reprogramming the local inflammatory environment through TNF/LT‐dependent signalling pathways.

## 2 Methods

Refer to Supplementary Methods section (Data S1).

## 3 Results

**FIGURE 1:**
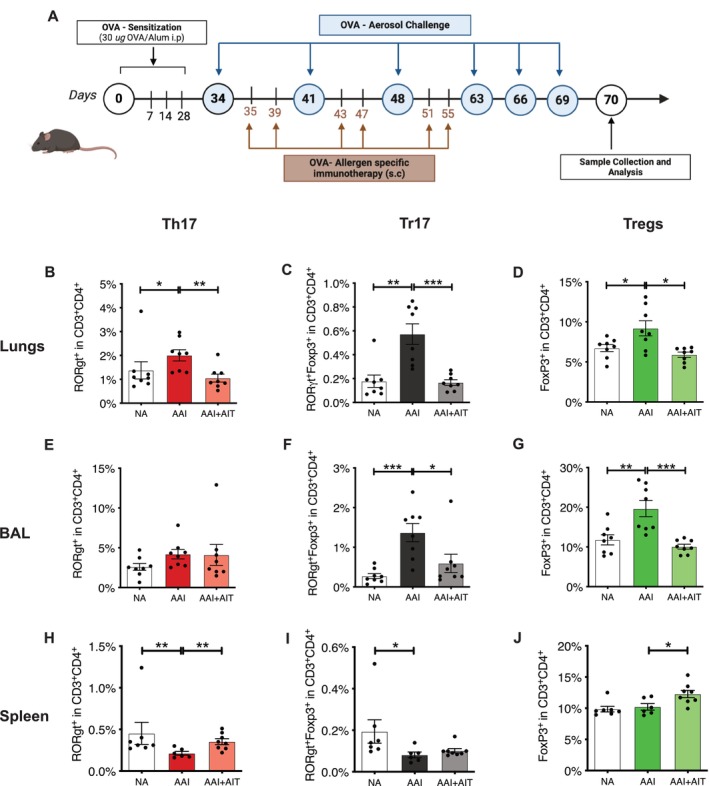
*Murine model of allergic airway inflammation and impact of AIT on Th17, Tr17 and Treg populations*. Schematic illustration of OVA‐induced murine model of AAI (A); Mice were sensitised on Days 0, 7, 14 and 28 followed by instillation challenges on Days 34, 41, 48, 63, 66 and 69 (blue arrows). Subcutaneous OVA AIT was performed on Days 35, 39, 51 and 55 (brown arrows) followed by analysis was performed on Day 70 (control *n* = 8; AAI *n* = 8; AAI + AIT *n* = 8). Representative flow cytometry plots (B) of frequency of RORgt and FOXP3 of CD4 + CD3+, CCR6+ live cells. Quantification of Th17 (Red), Tr17 (black) and Treg (Green) populations in lungs (C, D and E), in bronchoalveolar lavage (BAL; F, G and H) and in spleen (I, J and K). Statistical analysis was performed using Mann–Whitney *U* test and the statistical significance is indicated by * *p* < 0.05, *p* < 0.01, ***p < 0.001. (NA, Non‐Allergic; AAI, Allergic Airway Inflammation; AIT, Allergen Immunotherapy). The data were not pooled (no. of experiments = 1).

To determine the effect of AIT on the dynamics of the Th17 and Treg population in allergic airway inflammation, we performed flow cytometry using an established OVA mouse model (Figure 1A) and human samples of the AIT clinical trial PACIFIC. The mouse model's characteristics of OVA‐specific Th2 and OVA‐specific IgE response along with AIT‐mediated suppression were previously described [16].

### 3.1 AIT Restores the Balance of Th17, Tr17 and Treg Populations in Allergic Airway Inflammation

We quantified the population of Th17, Tr17 and Tregs locally in lung suspension cells and bronchoalveolar lavage (BAL) (Figure S1) Here, we observed that allergic airway inflammation significantly increased Th17, Tr17 and Treg populations in the lungs (Figure 1B–D). Even though a similar trend was observed in BAL, only Tr17 and Treg populations reached statistical significance (Figure 1E–G). In contrast, this inflammation‐mediated increase in Th17 and Tr17 cell frequency was not observed in the spleen. Instead, they were significantly decreased (Figure 1H,I), while the Tregs remained unchanged (Figure 1J). Notably, AIT decreased the Th17, Tr17 and Tregs population to levels nearly comparable to non‐allergic controls in the lungs and BAL (Figure 1B–G). On the contrary, Th17, Tr17 and Treg populations in the spleen were markedly reduced following allergic airway inflammation in comparison to healthy controls and they were significantly increased following AIT (Figure 1H–J). Collectively, these results indicate that AIT restores the local immune balance in the airways while also promoting systemic rebalance, emphasising its role as a potent modulator of local immune responses.

### 3.2 AIT Restores Th17 Cell Functionality and Enhances Proliferative Responses in Tr17 and Treg Cells

**FIGURE 2:**
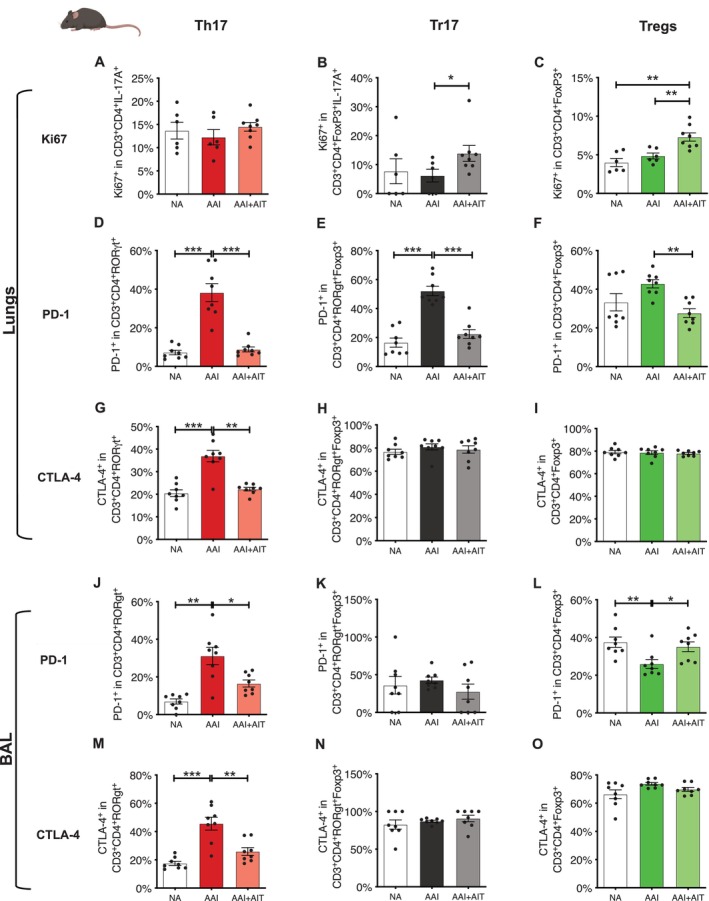
*Modulation of Th17, Tr17 and Treg cell exhaustion and proliferation by AIT*. Flow cytometric analysis of Th17, Tr17, and Treg populations in Lungs (A–I) and in BAL (J–O) in mice with and without AIT (Control *n* = 8; AAI *n* = 8; AAI + AIT *n* = 8). Quantification of Ki67+ proliferating Th17 (A), Tr17 (B) and Treg (C) cells in lungs. Quantification of exhaustion markers PD1 and CTLA4: PD‐1+ Th17 (D, J), Tr17 (E, K) and Treg (F, L) cells in lungs and BAL respectively; CTLA4+ Th17 (G, M), Tr17 (H, N) and Treg (I, O) cells in lungs and BAL respectively. Statistical analysis was performed using the Mann–Whitney U test, and the statistical significance is indicated by * *p* < 0.05, **p < 0.01, ***p < 0.001. The data were not pooled (no. of experiments = 1).

To assess the proliferative capacity of different T‐cell populations before and after AIT, Ki67+ Th17, Tr17 and Treg cells were quantified. A significant increase in Ki67+ proliferating Tr17 and Tregs was observed following AIT, but not in the Th17 population (Figure 2A–C). PD‐1+ and CTLA‐4+ Th17, Tr17 and Treg cells were quantified in both lungs and BAL to assess activation and exhaustion status (Figure 2D–O).

In allergic mice, PD‐1^+^ Th17, Tr17 and Treg cells were significantly increased in the lungs compared with non‐allergic controls (Figure 2D–F). CTLA‐4^+^ Th17 cells were also elevated (Figure 2G), whereas CTLA‐4^+^ Tr17 and Treg populations remained unchanged (Figure 2H,I). Following AIT, PD‐1^+^ expression in all three cell types decreased to near non‐allergic levels (Figure 2D–F), while CTLA‐4^+^ expression was significantly reduced only in Th17 cells (Figure 2G).

In BAL, PD‐1^+^ and CTLA‐4^+^ Th17 cells were similarly increased in allergic mice (Figure 2J,M), whereas Tr17 levels remained unchanged (Figure 2K,N). PD‐1^+^ Tregs were reduced in allergic mice (Figure 2L), while CTLA‐4^+^ Tregs were unaffected (Figure 2O). AIT significantly reduced PD‐1^+^ and CTLA‐4^+^ expression in Th17 cells (Figure 2J,M), but not in Tr17 or Treg populations (Figure 2K–O). A similar pattern was observed in the spleen (Figure S2A–E).

Together, these findings suggest that AIT selectively reverses the exhaustion‐like phenotype in Th17 cells while enhancing the proliferative capacity of Tr17 and Treg populations, contributing to the restoration of immune tolerance in the local microenvironment.

### 3.3 AIT Restores Mucosal Immune Balance but Does Not Reverse Circulating Th17 Functional Impairment

To assess the impact of AIT on Th17‐associated immune populations, sputum samples from AR and asthmatic patients were analysed before and after treatment. Sensitivity analysis indicated that age and sex did not significantly influence Th17 frequencies (age: *β* = 0.015, 95% CI −0.013 to 0.043, *p* = 0.29; sex: *β* = −0.091, 95% CI −0.395 to 0.213, *p* = 0.55). Predicted Th17 values at the median age (24 years) were comparable between sexes (0.76% vs. 0.69%), indicating minimal confounding. Sputum data were therefore analysed cross‐sectionally across healthy controls (HC), AR patients without AIT and AR patients receiving AIT (*n* = 20/6/10; supplementary methods [Data S1] and Table S1).

**FIGURE 3:**
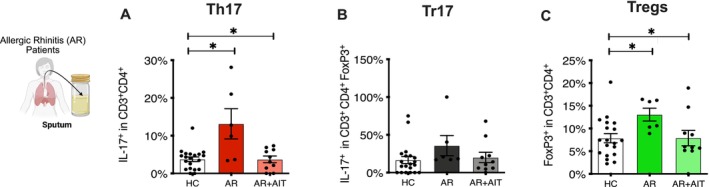
*Effect of AIT on Th17, Tr17 and Treg populations in the sputum of Allergic Rhinitis patients*. Flow cytometric quantification of Th17 (Figure 3A), Tr17 (Figure 3B), Treg (Figure 3C) populations in the sputum samples of healthy controls (*n* = 20) and allergic rhinitis patients before (*n* = 6) and after AIT (*n* = 10). Statistical analysis was performed using the Mann–Whitney *U* test, and the statistical significance is indicated by *p < 0.05, **p < 0.01, ***p < 0.001. The data were not pooled (no. of experiments = 1). Analyses are cross‐sectional; see Supplementary Methods (‘Sputum sample collection’ Data S1) and Table S1 for cohort details.

Flow cytometric analysis (Figure S3) revealed significantly elevated frequencies of Th17, Tr17 and Treg cells in AR patients compared with healthy controls (Figure 3A–C). Following AIT, these populations were significantly reduced, returning to levels comparable to healthy individuals. This normalisation was not observed in asthmatic patients (Figure S4), suggesting that AIT restores mucosal immune equilibrium in AR but not in asthma.

We next examined circulating Th17 cells using longitudinal blood samples collected before, during and after AIT. Flow cytometry revealed dynamic changes in cytokine production and activation markers (Figure S5A and Figure S6). AIT significantly increased IL‐17A^+^ Th17 cells during the top‐dose phase and in follow‐up seasons (Figure S5B). In contrast, PD‐1^+^ Th17 and IL‐2^+^PD‐1^+^ Th17 cell frequencies remained unchanged (Figure S5C,D). CTLA‐4^+^ Th17 cells significantly increased at T9 in the final follow‐up season (Figure S5E), whereas IL‐2^+^CTLA‐4^+^ Th17 cells decreased at the same time point (Figure S5F). Additional subsets associated with anergy‐like states, including TCF1^+^PD‐1^+^ Th17, TCF1^+^CTLA‐4^+^ Th17 and PD‐1^+^CTLA‐4^+^ Th17 cells, remained stable throughout treatment (Figure S7A–C).

To determine whether checkpoint blockade could restore Th17 effector function, peripheral blood cells were stimulated with anti‐CD3 and anti‐CD28 in the presence of the anti‐PD‐1 antibody Nivolumab or an IgG control. PD‐1 blockade did not alter IL‐2^+^ Th17 or IL‐2^+^PD‐1^+^ Th17 frequencies, which remained comparable between conditions (Figure S5G,H).

Together, these findings indicate that AIT restores mucosal Th17‐associated immune balance in AR but does not reverse the unresponsive phenotype of circulating Th17 cells, even upon PD‐1 blockade.

### 3.4 AIT‐Driven Trans‐Differentiation Mediated by Tr17 Cells

**FIGURE 4:**
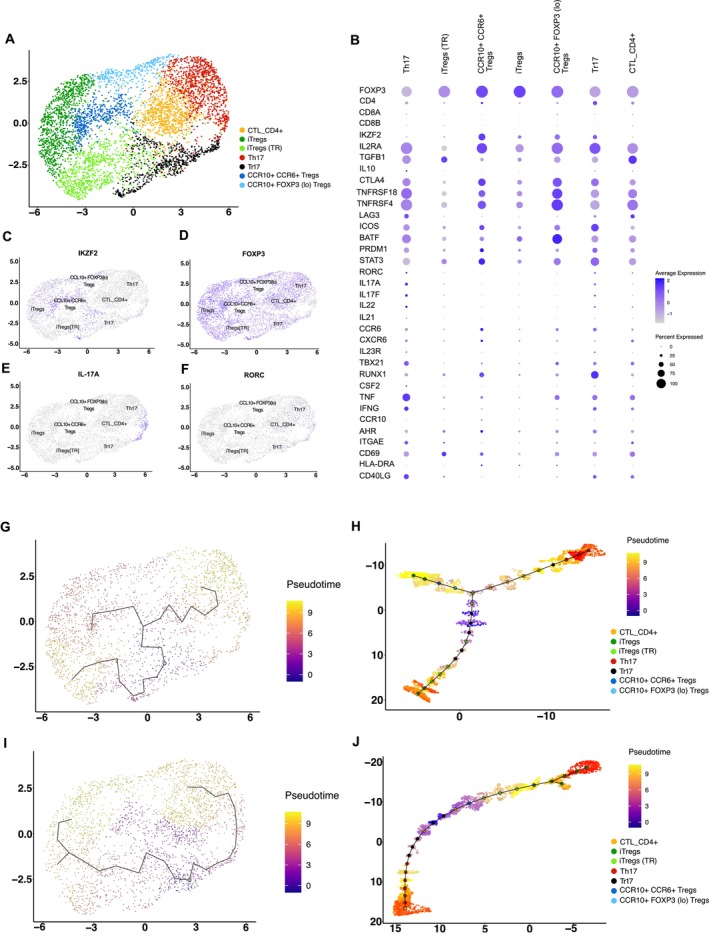
*AIT‐Driven Trans‐differentiation Mediated by Tr17 Cells*. (A) Single‐cell mRNA sequencing analysis of PBMCs derived from the blood samples of PACIFIC cohort at baseline (T0) before AIT and after the AIT during the maintenance phase (T6). Data were analysed using Louvain cluster analysis, with cell identification performed by the index genes for Th17, Tr17, iTregs, iTregs (TR), *CCL10+ CCR6+* Tregs, *CCL10+ FOXP3(lo)* Tregs and CTL‐CD4+ cells. The cellular identity in the UMAP clustering is shown with the following cell types: CTL‐CD4+ (yellow), iTregs (dark green), iTregs (TR) (light green), Th17 (red), Tr17 (black), *CCL10+ CCR6+* Tregs (dark blue) and *CCL10+ FOXP3(lo)* Tregs (light blue). (B) The index genes and their average expression intensity. The proportion of cells expressing the respective gene is represented by the size of the circles. UMAP of cell frequency expressing the index genes *IL17A* (C), *RORC* (D), *IKZF2* (E) and *FOXP3* (F). Transitional representation of cell populations along a pseudotime trajectory generated with Monocle (G–J): UMAP visualisation of single‐lineage cells before AIT at T0 (G) and after AIT at T6 (I) marked with inferred pseudotime. The black curve denotes the inferred lineage. The respective visualisation of bifurcating cells marked with inferred pseudotime before AIT at T0 (H) and after AIT at T6 (J). The black curve denotes the inferred lineage, and the different T‐cell populations are denoted on the black line in their respective colour demotion.

Single‐cell mRNA sequencing of *CCR6+*, *FOXP3+*, and/or *IL‐17A +* T cells from the PACIFIC cohort: collected at baseline (T0) and following 1 year of AIT during the maintenance phase (T6) revealed distinct immune cell subsets via Louvain clustering such as Th17, Tr17, induced Tregs (iTregs), iTregs (TR), *CCL10+ CCR6+* Tregs, *CCL10+ FOXP3(lo)* Tregs, and CTL‐CD4+ cells (Figure 4A,B). We defined the Th17, Tr17, and Treg population using their respective index genes of the three populations: *IL17A, RORC, IKZF2*, and *FOXP3*, and assigned their origin to specific and separate cell compartments (Figure 4C–F). Developmental pseudotime trajectory analysis revealed potential developmental relationships among the identified *CCR6+* T‐cell populations, which were mediated by AIT. UMAP projection coloured by pseudotime (Figure 4G,I) visualised a continuous developmental trajectory linking multiple cell states, with the trajectory backbone (black lines) delineating potential differentiation pathways. Under control conditions (Figure 4H), Tr17 cells were positioned in close proximity to Th17 cells, indicating a more pro‐inflammatory phenotype in the absence of treatment. A shift in the cellular trajectories was observed following AIT (Figure 4J), positioning Tr17 cells to a more central position between Th17 and iTregs, potentially indicating that AIT may drive a trans‐differentiation process in which Tr17 cells assume an intermediary state.

### 3.5 Notable Alterations in Receptor‐Ligand‐Mediated Communications Between Different T‐Cell Types Before and After AIT

**FIGURE 5:**
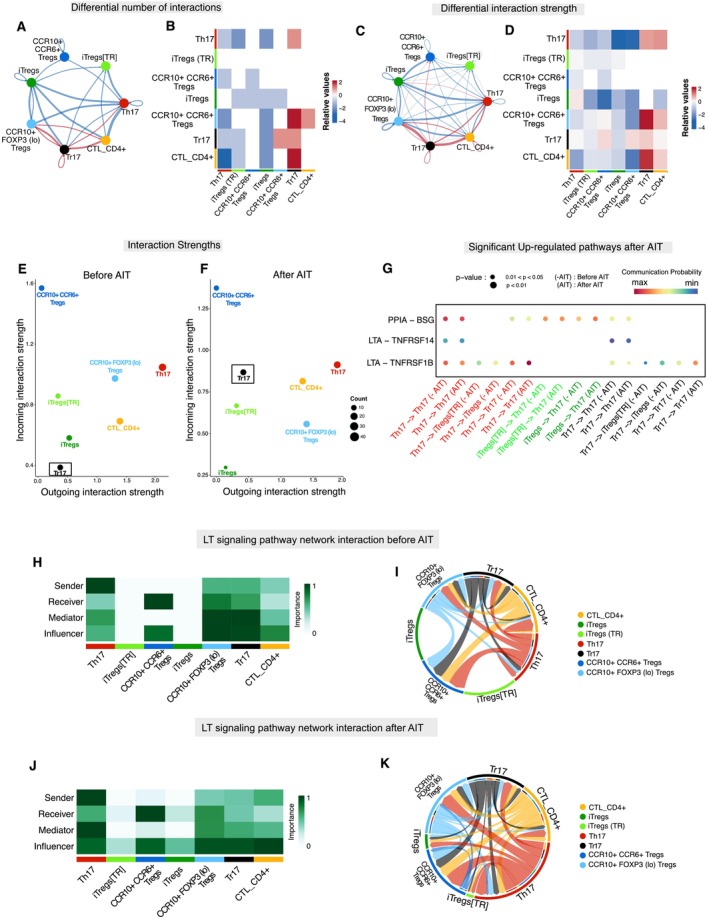
*Notable alterations in receptor‐ligand‐mediated communications between different T‐cell types before and after AIT*. Circle plots depicting the interaction numbers (A) and interaction strength (C) between different T‐cell types. Blue lines indicate that the displayed communication is decreased, while red lines indicate that communication is increased. The arrows indicate the direction of intercellular communication. The same data of interaction numbers (B) and interaction strength (D) between different T‐cell types visualised by heatmaps for annotation. Scatter plot showing the intensity of the outgoing and incoming interactions in a two‐dimensional manifold of different T‐cell populations before AIT at T0 (E) and after AIT at T6 (F). The size of the circles suggests the numbers of significantly expressed receptor‐ligand pathways of different T‐cell populations. (G) Comparison of the significant pathways before and after AIT, which contribute to the signalling between the populations of interest: Th17, iTregs, iTregs (TR) and Tr17 subpopulations. Dot colour reflects communication probabilities and dot size represents computed *p*‐values. Empty space means the communication probability is zero. *p*‐values are computed from one‐sided permutation test. Heatmap shows the relative importance of each cell type as sender, receiver, mediator and influencer in the LT Pathway based on the computed four network centrality measures of different signalling pathways (H and J). Chord diagram for visualising cell–cell communication through LT signalling pathway before AIT at T0 (I) and after AIT at T6 (K). The lines represent changes in ligand‐receptor interaction strengths and the colour bars in the inner circles indicate targeting cell types of the outgoing signalling while noncolour part for incoming signalling.

To investigate the functional implications of the shift observed in the trajectory analysis, cell–cell communication network analysis was performed in both conditions: Before (T0) and After AIT (T6). This analysis identified differences in the number, strength and directionality of interactions between various cell subsets. Network visualisation showed differences in interaction numbers and strengths among all identified subsets, especially Tr17 cells exhibited increased connectivity following AIT (Figure 5A–D).

**FIGURE 6:**
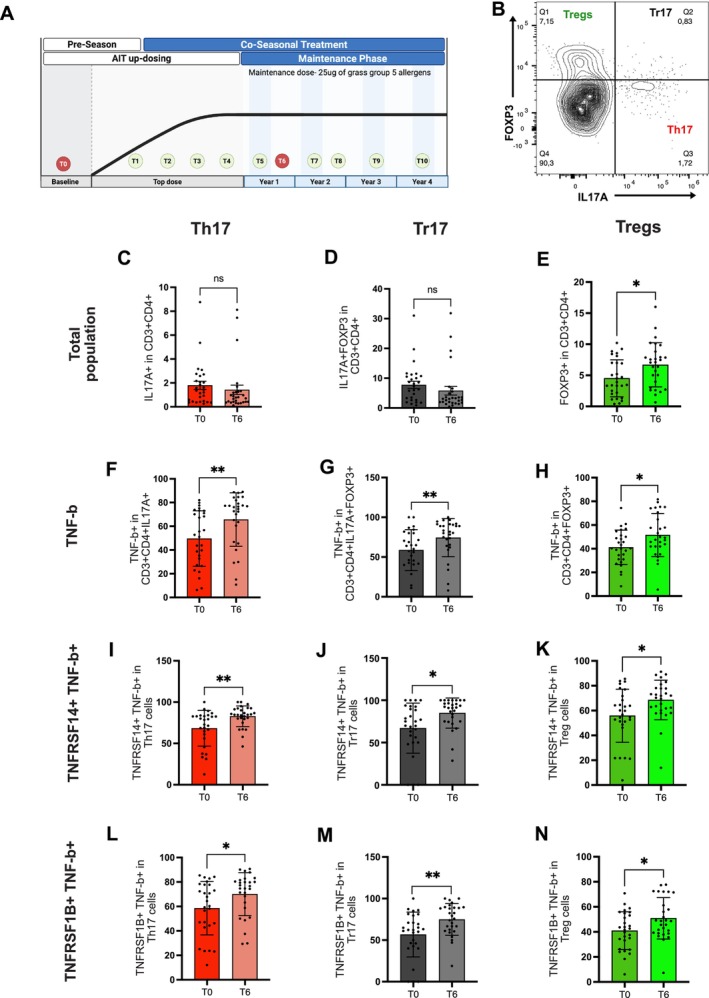
*AIT is associated with enhanced TNFR‐LT‐α axis engagement*. Schematic overview of the sample collection timeline in the grass pollen AIT study (A). Representative flow cytometry plots (*n* = 28) and quantification of Th17, Tr17 and Treg subset frequencies at baseline (T0) and after 1 year of AIT (T6) (B–E). Frequencies of *LT‐α* ^+^ single‐positive cells and HVEM^+^/TNFR2^+^ *LT‐α* ^+^ double‐positive cells within Th17, Tr17 and Treg subsets (F–N). Statistical analysis was performed using Mann–Whitney *U* test and the statistical significance is indicated by *p < 0.05, **p < 0.01, ***p < 0.001. Data represent a single experiment.

Distinctly, the scatter plot (Figure 5E,F) revealed a notable increase in both incoming and outgoing signals of only the Tr17 cells following AIT. Differential expression analysis (DEA) identified various upregulated signalling pathways across different T‐cell populations, including *MIF, LT, CypA, TNF, LIGHT* and *CCL*. These signalling patterns were substantially mediated between Th17 and Tr17 cell populations which were altered following AIT (Figure S8A–D). Among these, only a few signalling pathways were significantly upregulated following AIT (*p* < 0.01) (Figure 6G). The *LTA‐TNFRSF1B* pathway had the highest communication probability, with the strongest interaction occurring between Th17 and Tr17 cells after AIT. The *LTA‐TNFRSF1B, LTA‐TNFRSF14* and *PPIA‐BSG* pathways were significantly upregulated and had a very high communication probability between the population of interest. Further analysis of Tr17 signalling patterns showed that members of the Lymphotoxin (*LT*) and TNF Tumour necrosis factor (*TNF*) pathways were key mediators of its interactions with other cell populations (Figure S8E–H). Notably, specific ligand‐receptor interactions such as *LTA, TNFRSF1B* and *TNFRSF14* were significantly upregulated both before and after AIT. The *LTA* ligand was strongly upregulated in iTregs after AIT, while the *TNFRSF1B* receptor expression increased significantly in Tr17 cells (Figure S8I). Network centrality analysis identified that Tr17 cell populations are the most prominent mediators through TNF signalling (Figure 5H–K and Figure S8J–M). These network analyses suggest that AIT not only alters cellular frequencies and functional states of cells but also reshapes intercellular communications.

### 3.6 AIT Is Associated With Enhanced TNFR‐LT‐α Axis Engagement

To validate our in silico findings from scRNA‐seq, we analysed peripheral blood samples from grass pollen allergic patients at baseline (T0) and after 1 year of AIT (T6) (Figure 6A). Flow cytometric profiling (Figure 6B, Figure S9 and Figure S12) revealed a selective modulation of TNF receptor signalling and effector cytokine expression across Th17, Tr17, and Treg subsets. Consistent with the scRNA‐seq data, AIT induced a selective upregulation of TNF‐receptor superfamily members and an increase in LT‐α (TNF‐β) producing cells across these subsets. After 1 year of treatment, we observed a clear upregulation of the Treg compartment, whereas total Th17 and Tr17 frequencies remained unchanged (Figure 6C–E). Concomitantly, LT‐α expression was significantly elevated in Th17, Tr17 and Treg populations (Figure 6F–H), and the proportion of HVEM^+^LT‐α^+^ and TNFR2^+^ LT‐α^+^ double‐positive cells increased in all three subsets (Figure 6I–N), validating the predicted LT‐α‐HVEM/TNFR2 ligand‐receptor interaction. However, while the individual receptor expression of TNFR2 (TNFRSF1B) remained unaltered following AIT (Figure S10A–C), HVEM (TNFRSF14) expression was downregulated in Tr17 and significantly so in Tregs yet remained unchanged in Th17 cells (Figure S10D–F). This observed trend in that of conventional Tregs remained the same as iTregs (Figure S11). Analysis of inhibitory and proliferation markers revealed that PD‐1 expression was significantly downregulated (Figure S10G–I), while CTLA‐4 expression increased across Th17, Tr17, and Treg populations but reached statistical significance only in Th17 and Tr17 cells (Figure S10J–L). IL‐2 production remained unchanged (Figure S10P–R), and Ki‐67 expression in these subsets was decreased following AIT (Figure S10M–O), consistent with reduced proliferative activity and a shift toward a less responsive or suppressed cellular state.

### 3.7 LT‐α‐Induced TNF Receptor Signalling Reveals Differential T‐Cell Responsiveness After AIT

**FIGURE 7:**
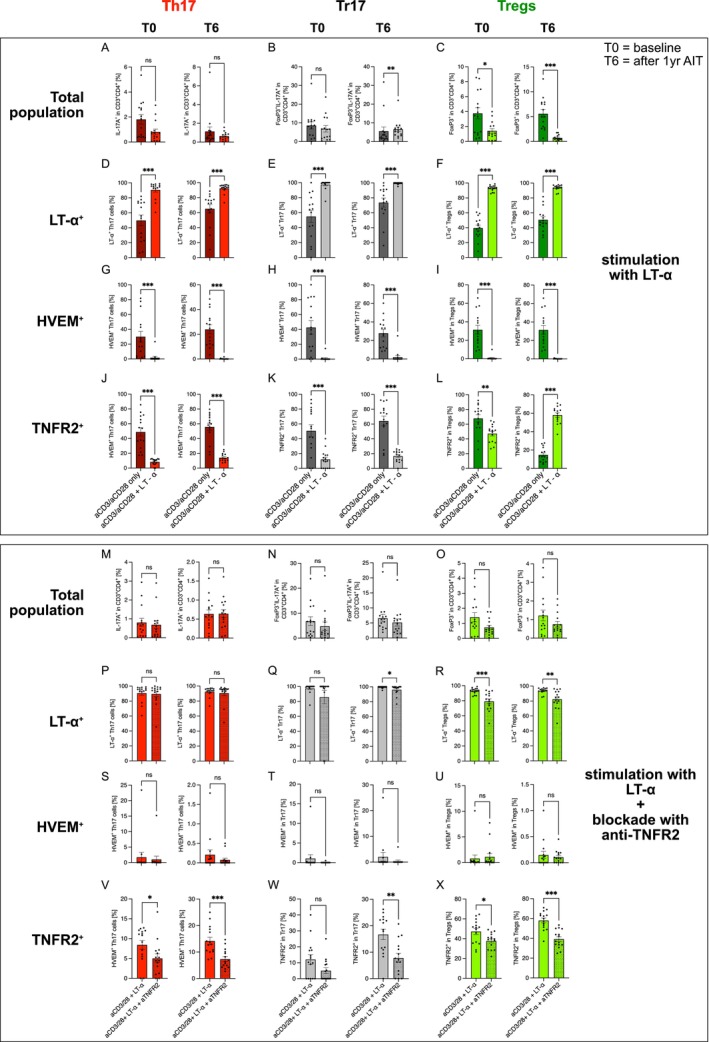
*LT‐α‐induced TNF receptor signalling reveals differential T‐cell responsiveness after AIT*. PBMCs from grass pollen‐allergic patients were stimulated with anti‐CD3/anti‐CD28 alone, anti‐CD3/anti‐CD28 + LT‐α or anti‐CD3/anti‐CD28 + LT‐α in the presence of a TNFR2‐blocking antibody at baseline (T0) and after 1 year of allergen immunotherapy (T6). Frequencies of total Th17, Tr17, and Treg populations are shown in A‐C. Frequencies of LT‐α^+^ (D–F), HVEM^+^ (G–I) and TNFR2^+^ (J–L) cells within the respective T‐cell subsets following LT‐α stimulation are shown. To assess the contribution of TNFR2 signalling, PBMCs were stimulated with anti‐CD3/anti‐CD28 + LT‐α in the presence or absence of a TNFR2‐blocking antibody. Frequencies of total Th17, Tr17 and Treg populations are shown in M–O, and frequencies of LT‐α^+^ (P–R), HVEM^+^ (S–U), and TNFR2^+^ (V–X) cells within the respective subsets are shown. In each subfigure, the left panels correspond to baseline samples (T0) and the right panels to samples obtained after 1 year of AIT (T6). Each dot represents one patient (*n* = 15). Statistical comparisons were performed using the two‐tailed Mann–Whitney *U* test; significance is indicated as *p < 0.05, **p < 0.01, ***p < 0.001. Data are presented as mean ± SEM.

To functionally validate the TNF receptor signalling axis, PBMCs from grass pollen‐allergic patients were stimulated with anti‐CD3/anti‐CD28 in combination with LT‐α, with or without TNFR2 blockade, at baseline (T0) and after 1 year of AIT (T6). LT‐α stimulation did not alter Th17, increased Tr17 frequencies only at T6 (Figure 7A,B), and reduced Treg frequencies at both T0 and T6 (Figure 7C), indicating altered regulatory T‐cell responsiveness following AIT.

LT‐α stimulation induced robust autocrine cytokine production across all subsets, reflected by increased LT‐α^+^ frequencies in Th17, Tr17, and Tregs (Figure 7D–F). In effector subsets, LT‐α stimulation was accompanied by pronounced downregulation of HVEM and TNFR2 (Figures 7G,H and 7J,K), consistent with ligand‐induced receptor modulation. In contrast, Tregs displayed increased TNFR2 expression at T6 (Figure 7L), suggesting enhanced receptor responsiveness after AIT.

To assess the contribution of TNFR2 signalling, PBMCs were stimulated with LT‐α in the presence or absence of a TNFR2‐blocking antibody. Total Th17, Tr17, and Treg frequencies remained unchanged under blockade (Figure 7M–O). LT‐α^+^ frequencies were largely preserved in Th17 cells but partially reduced in Tr17 and particularly in Tregs under TNFR2 inhibition (Figure 7P–R). HVEM expression was unaffected by TNFR2 blockade (Figure 7S–U), whereas TNFR2^+^ frequencies decreased across subsets, most prominently in Tregs (Figure 7V–X), confirming effective pathway inhibition.

Further analysis of receptor‐cytokine co‐expression confirmed subset‐specific responses to LT‐α stimulation. TNFR2^+^LT‐α^+^ cells increased particularly in Tregs, whereas HVEM^+^LT‐α^+^ effector populations decreased (Figure S13). Functional profiling revealed reduced PD‐1 and Ki‐67 together with increased CTLA‐4 and IL‐2 expression across subsets (Figure S13). The CTLA‐4 increase in Tregs was partially attenuated by TNFR2 blockade (Figure S14), indicating partial TNFR2 dependence of the observed regulatory programme.

## 4 Discussion

In a previous study, we demonstrated that peripheral frequencies of Th17 and Tr17 cells correlate with the clinical success of AIT after 3 years [13]. In this study, we demonstrate that AIT restores the equilibrium between Th17 and Treg cells at the site of allergic inflammation and provide evidence that TNF signalling contributes to AIT‐induced immune modulation, while transitory Tr17 cells emerge as pivotal regulators steering the immune response toward tolerance.

The mouse model reproduced key features of allergic airway inflammation, including altered Th17, Tr17 and Treg distributions between the lungs, BAL and the spleen. AIT normalised Th17, Tr17 and Treg frequencies in the lungs and BAL while increasing splenic Treg populations, suggesting a restoration of local immune balance together with enhanced systemic regulatory responses. These findings align with previous studies in similar mouse models reporting increased Th17 and Treg frequencies and elevated IL‐4 and IL‐17 levels in the lungs and BAL following allergic airway inflammation [17, 18, 19]. These effects subsequently returned to baseline following AIT, while the level of IL‐10 was significantly elevated only post AIT. This highlights the ability of AIT to reverse local inflammation and exert systemic regulatory responses.

Differential responses were observed among T‐cell subsets, with AIT markedly enhancing the proliferative capacity of both Tr17 and Treg cells, while Th17 cells remained unresponsive. Concurrently, upregulated PD‐1 and CTLA‐4 on Th17 cells indicate that local immune activation induces an exhaustion‐like state. PD‐1 expression is known to be rapidly induced following TCR engagement, while its co‐expression with CTLA‐4 indicates exhaustion [20, 21]. These markers were effectively reduced by AIT in local compartments yet remained unaltered in the spleen; findings that imply AIT selectively reconditions Th17 exhaustion at the site of inflammation [19, 22]. This observation is parallel to findings in other T‐cell subsets, such as Th2 cells, where AIT induced an upregulation of PD‐1 which was associated with enhanced cellular exhaustion [16, 19]. In addition, Wang et al. demonstrated that AIT attenuates Th2‐driven responses by reducing Th2 cytokine production, eosinophilic inflammation and allergen‐specific IgE, while increasing exhaustion marker expression (PD‐1, CTLA‐4) on Th2 cells in local lung tissue [16]. Together with our observations on Th17, Tr17 and Treg populations, these data suggest that AIT restores immune balance not only through regulatory and Th17 modulation but also by dampening Th2‐mediated inflammation. The clinical relevance of our findings is further translational by our analysis of human sputum from AR patients, who also demonstrated elevated frequencies of Th17, Tr17 and Treg cells, which were notably reduced following AIT. Unexpectedly, this pattern, however, was not observed in the sputum of asthmatic patients. Whether this effect is due to the chronic nature of asthma needs to be a subject of future studies. A limitation is the cross‐sectional design and the relatively small subgroup, which precluded robust multivariable adjustment; therefore, only non‐parametric group comparisons are reported.

A key observation was the progressive accumulation of exhaustion‐associated circulating Th17 cells during the three‐year AIT period, reflected by increased CTLA‐4 and reduced IL‐2 expression. Consistently, in vitro PD‐1 blockade with Nivolumab failed to restore IL‐2 production, suggesting a persistent dysfunctional phenotype of circulating Th17 cells [23, 24]. These findings indicate that, despite effective local immune reprogramming by AIT, systemic Th17 dysfunction may persist and warrants further investigation.

Single‐cell transcriptomics of patients' peripheral blood before and after 1 year of AIT revealed cellular heterogeneity, identifying Th17, Tr17 and Treg clusters, as well as novel subsets like *CCR10* ^+^CCR6^+^ Tregs, *CCR10* ^+^FOXP3^lo^ Tregs and CTL‐CD4^+^ cells [25, 26, 27]. For the first time at the single‐cell level, we confirmed a Tr17 population co‐expressing *IL‐17A* and *FOXP3* [12, 28]. Furthermore, pseudotime trajectory analysis establishes a dynamic relationship between the subsets. At baseline, Tr17 cells clustered with Th17 cells, reflecting pro‐inflammatory features. Following AIT, they shifted centrally between Th17 and iTregs, implying increased plasticity and a transitional role in reprogramming tolerance.

In particular through the cell–cell communication network, we demonstrate that AIT significantly reshapes the intercellular signalling network, with the Tr17 population playing a central role. Notably, the TNF signalling pathway emerged as a critical mediator, with *LTA‐TNFRSF1B* exhibiting the highest communication probability and complementing the observed phenotypic shifts. TNF signalling contributes to allergic inflammation through LT‐α‐HVEM interactions that promote Th2‐skewed immune responses [29, 30, 31]. In contrast, LT‐α engagement of TNFR2 induces regulatory T‐cell function by stabilising FOXP3 and enhancing suppressive activity [32, 33, 34, 35]. Consistent with this, after 1 year of treatment, our data indicate a shift toward regulatory dominance and reduced effector activity. This is reflected in increased LT‐α/TNFR2‐associated signalling, reduced HVEM expression particularly within effector subsets and decreased PD‐1 and Ki‐67 expression together with increased IL‐2 production. Across Treg, Tr17, and Th17 subsets, these changes were consistent with a shift toward regulatory signalling and reduced effector activity. Th17 and Tr17 cells showed features of a hyporesponsive effector compartment, potentially reinforced by CTLA‐4‐mediated suppression. These findings align with reports that LT‐α/TNFR2 signalling dampens Th17 responses and suppresses RORγt expression, thereby mitigating allergic inflammation [36, 37].

Ex vivo LT‐α stimulation and TNFR2 blockade demonstrate that AIT reshapes not only the magnitude but also the quality of TNF responses. LT‐α induced a strong autocrine signalling loop across T‐cell subsets, while TNFR2 signalling contributed particularly to LT‐α‐responsive Tregs. These findings indicate that AIT fine‐tunes TNF receptor sensitivity and promotes a suppressive programme characterised by increased CTLA‐4 expression and reduced proliferative activity.

Our data suggest that 1 year of grass pollen AIT promotes regulatory immunity while attenuating effector T‐cell activity through LT‐α/TNFR2‐associated signalling. Future studies should define the cellular hierarchy of TNF‐mediated communication and dissect TNFR1 versus TNFR2 downstream pathways in sorted subsets.

In summary, our study identifies an LT‐α‐driven autocrine signalling programme that reshapes the Th17‐Tr17‐Treg axis during AIT. While LT‐α is broadly produced across CD4^+^ T‐cell subsets, TNFR2 signalling selectively supports regulatory responses in Tregs and is accompanied by increased CTLA‐4 expression and reduced proliferative activity, consistent with a suppressive immune phenotype. These findings suggest that AIT promotes immune tolerance by fine‐tuning TNF receptor sensitivity rather than merely expanding regulatory populations, highlighting LT‐α‐TNFR2 signalling as a potential target to enhance clinical responses to AIT and promote sustained immune tolerance.

## Author Contributions

H.S.C., A.A.G., A.M.C., C.A.J., C.B.S.‐W. and S.B. designed the study. H.S.C., A.H., F.A., F.G., J.K., M.O., M.P., L.P., D.P., L.S.z.B., S.H., S.‐H.W., U.M.Z. and S.K. were involved in experiments execution. C.O., C.A.J. and C.B.S.‐W., were involved in the ethics of the study and critical review of the manuscript. H.S.C. and A.A.G. analysed the data. H.S.C., A.A.G., C.A.J. and C.B.S.‐W., interpreted the data. H.S.C. did the literature search. All authors wrote the manuscript.

## Funding

This study was supported by the German Center for Lung Research (DZL) to CSW, by grants from Deutsche Forschungsgemeinschaft RTG2668 (Project A5, Project‐ID: 435874434) to CSW, and by grants from Else‐Kröner‐Fresenius‐Stiftung (CASSAVA project; 2024_EKEA.137) to CAJ.

## Conflicts of Interest

The authors declare the following competing interests: Prof. Dr. Zissler reports grants from Federal Ministry of Education and Research (BMBF) for the German Center for Lung Research, (DZL), grants and personal fees from German Research Foundation (DFG Grant No. 398577603), grants and personal fees from Bavarian State Office for Health and Food Safety (K1‐2497‐GLB‐20‐V4), during the conduct of the study; grants from European Institute of Technology (EIT), grants from CFi‐aktiv e.V. ukoviszidose Selbsthilfe Südbayern outside the submitted work. In addition, he reports personal fees from Max Aicher foundation for his endowed chair for building biology, airway and indoor health as well as grants and personal fees from the Bavarian States Ministry of Science and Art outside the submitted work. Dr. Kotz reports grants for a travelling scholarship by the German ENT society (DGHNO), speaker honoraria, consultancy or advisory fees and/or research support and other, all via Technical University of Munich from GSK, HAL Allergy, and Biocryst, all outside this submitted work. Dr. zur Bonsen received speaking honoraria from AbbVie and Novartis, all outside of this submitted work. Prof. Dr. Blank has received honorarium for talks or research grants from Thermo Fischer Scientific Inc., Bencard Allergie GmbH, Allergopharma GmbH & Co. KG, LETI Pharma GmbH and Allergy Therapeutics PLC, all outside the submitted work. PD Dr. Chaker reports grants from German Center for Lung Research (DZL) during the conduct of the study; grants, speaker honoraria, consultancy or advisory fees and/or research support and other, all via Technical University of Munich from ALK‐Abello, AstraZeneca, Bencard/Allergen Therapeutics, ASIT Biotech, GSK, Hippo Dx, Novartis, LETI, Roche, Sanofi, Regeneron, Zeller, grants from Federal German Ministry of Education and Research, grants from European Institute of Technology, all outside this submitted work. Prof. Dr. Schmidt‐Weber reports grants from Federal Ministry of Research and Education, during the conduct of the study and personal fees from Zeller AG, during the conduct of the study; grants from Allergopharma and personal fees from Sanofi, Leti Pharma, Aimmune, outside the submitted work. PD Dr. Jakwerth reports grants from DFG—Excellence University Strategy (EXU) at International Graduate School of Science and Engineering (IGSSE) (Imperial‐TUM (Technical University Munich) Joint Academy of Doctoral Studies (JADS); PANORAMA project), Deutsche Forschungsgemeinschaft RTG2668 (Project A1, Project‐ID: 435874434), grants from Zeller AG, outside the submitted work. Ms. Charles, Dr. Gabr, Dr. Wang, Dr. Heine, Dr. Hills, Dr. Pogorolev, Dr. Heldner, Ms. Pechtold, Dr. Kau, Mr. Guerth, Ms. Oelsner, PD. Dr. Ohnmacht and Prof. Dr. Alessandrini, Dr. Plaschke have nothing to disclose. AI‐based tools were used for parts of the scRNA‐seq data analysis, and Grammarly was used for language editing. All analyses and interpretations were performed and verified by the authors.

## Supporting information


**Data S1:** all70367‐sup‐0001‐Supinfo1.docx.

**Table S1:** Characteristics of allergic rhinitis and allergic asthmatic patients with without allergen immunotherapy from whom sputum was collected.
**Table S2:** Characteristics of grass pollen‐allergic patients with immunotherapy (PACIFIC study).
**Table S3:** Monoclonal antibodies used in flow cytometry analyses.

**Table S4:** Single cell sequencing cluster identification markers.

**Figure S1:** Representative sequential gating strategy of OVA Mice model with allergic airway inflammation (AAI) and AAI treated with allergen immunotherapy (AIT).
**Figure S2:** PD‐1 and CTLA‐4 Expression in Th17, Tr17 and Treg Populations in the Spleen.
**Figure S3:** Representative sequential gating strategy of sputum samples of allergic rhinitis and allergic asthmatic patients with and without AIT.
**Figure S4:** Effect of AIT on Th17, Tr17 and Treg populations in the sputum of Asthmatic patients.
**Figure S5:** Modulation of Th17 Cell Phenotype in Blood Following AIT.
**Figure S6:** Representative sequential gating strategy on the PBMCs isolated from allergic patients through the course of AIT.
**Figure S7:** Role of AIT on the stemness of exhausted Th17 Cell Phenotype in blood.
**Figure S8:** Notable alterations in receptors‐ligands‐mediated communications between different cell types before and after AIT.
**Figure S9:** Representative sequential gating strategy on the PBMCs isolated from grass‐pollen allergic patients before and after 1 year of AIT.
**Figure S10:**. Checkpoint and proliferation markers are largely unchanged after 1 year of AIT.
**Figure S11:**. TNFR‐LT‐α marker expression in iTregs after 1 year of AIT.
**Figure S12:** Representative staining controls for PACIFIC Cohort TNF Pathway Profiling flow cytometry panel involving appropriate FMO and Single stain layover for individual markers.
**Figure S13:**. Subset‐dependent changes in TNF signalling following LT‐α stimulation after 1 year of AIT.
**Figure S14:** TNFR2 blockade attenuates LT‐α‐induced signalling and checkpoint remodelling across T‐cell subsets.

## Data Availability

The data that support the findings of this study are openly available in Gene Expression Omnibus (GEO) at https://www.ncbi.nlm.nih.gov/geo/.
