# Supplementary material for: TNF Pathway‐Mediated Tolerogenic T‐Cell Trajectory Driven by Allergen Immunotherapy
Source: Allergy. 2026 Apr 30;81(6):2184–97. doi: 10.1111/all.70367 (PMC13256266; doi:10.1111/all.70367)
Supplement: Supplementary file 1 — Data S1: all70367‐sup‐0001‐Supinfo1.docx. [file ALL-81-2184-s004.docx]

**TNF pathway-mediated tolerogenic T cell trajectory driven by allergen immunotherapy**

**Authors:** Helen S. Charles^1,2^, M.Sc, Amr A. Gabr^1,2,3^, Ph.D., Shu-Hung Wang^1^, M.D. Ph.D., Ulrich M. Zissler ^1,2^, Ph.D., Sonja Heine^1^, Ph.D, Alexander Heldner^1^, Ph.D., Sebastian Kotz^4^, M.D., Lisa Pechtold^4^, M.D., Lynn S. zur Bonsen^4^, MD, Dimitrii Pogorelov^1^; Ph.D., Josephine Kau^4^, M.D., Mirjam Plaschke^4^, M.D., Miriam Hills^5^, Ph.D., Ferdinand Guerth^1^, M.Sc., Madlen Oelsner^1^, Caspar Ohnmacht^1^, Ph.D., Francesca Alessandrini^1^, Ph.D., Simon Blank^1^, Ph.D., Adam M. Chaker^1,4^, M.D., Carsten B. Schmidt-Weber^1,2^, Ph.D., Constanze A. Jakwerth^1,2,*^, Ph.D.

**Affiliations:**

^1^ Center of Allergy and Environment (ZAUM), School of Medicine and Health, Technical University of Munich, and Helmholtz Munich, Munich, Germany

^2^ Member of the German Center for Lung Research (DZL), Germany

^3^ Department of Physiology, Faculty of Veterinary Medicine, Cairo University, Giza 12211, Egypt

^4^ Department of Otorhinolaryngology, TUM School of Medicine and Health, Klinikum rechts der Isar, Technical University of Munich, Munich, Germany

^5^ Department of Dermatology and Allergy Biederstein, School of Medicine, Technical University of Munich, Munich, Germany

*** Corresponding author:**

PD. Dr. Constanze A. Jakwerth

Center of Allergy and Environment (ZAUM),

Technische Universität and Helmholtz Center Munich,

Biedersteiner Str. 29, 80802 München, Germany

[constanze.jakwerth@tum.de](mailto:constanze.jakwerth@tum.de)

Phone: +49 89 41403472

**SUPPLEMENTARY METHODS**

**Mice**

Six-week-old, female SPF-free C57BL/6J mice, purchased from Charles River (Sulzfeld, Germany) were provided with food and water *ad libitum*. The experiment was carried out in accordance with German federal regulations regarding the care and use of laboratory animals. Approval for the study was granted by the Government of the District of Upper Bavaria and the Animal Care and Use Committee of the Helmholtz Center Munich (55.2-1-54-2532-50-2017). Biosamples were taken from the study described by Wang et al (PMID: 33969495) following the protocol of Russkamp et al (30829405)

**Ovalbumin immunotherapy model**

The mouse model in this study has been previously described in detail (19, 34) the same treatment protocols and organ harvesting methods outlined there were applied in this study. Mice were assigned to three different groups based on treatment conditions: Non-Allergic (NA), Allergic Airway Inflammation (AAI), and AAI with Allergen Immunotherapy (AAI+AIT). On days 0, 7, 14, and 28, intraperitoneal injections of either 30 μg ovalbumin (Grade V, Sigma-Aldrich, Merck, Darmstadt, Germany) along with 2 mg aluminum hydroxide (ImjectAlum, Thermo Fisher Scientific, Waltham, MA, USA) or only 2 mg aluminum hydroxide in 200 μl PBS were given to sensitise the allergic and non-allergic groups of mice, respectively. Subcutaneous injections of OVA (500 μg/ 200 μl PBS) were given to the AIT group, whereas the AAI and NA group received a sham treatment with 200 μl PBS on days 35, 39, 43, 47, 51, and 55. During the immunotherapy phase, all mice were exposed to 1% nebulised ovalbumin for 15 minutes in a challenge chamber on days 34, 41, and 48 and again towards the end of the experiment on days 63, 66, and 69. Finally, all mice were euthanised on day 70, and analysis was performed.

**Patients and Blood Samples**

Peripheral blood mononuclear cells (PBMCs) from a cross-sectional patient group were obtained. The Prospective Allergy and Clinical Immune Function Cohort study (PACIFIC, EudraCT 2015–003545-25) has been previously described (13). The study involved 40 patients suffering from grass pollen allergies who fulfilled the ARIA criteria for moderate-severe chronic allergic rhinitis during the grass pollen season. Inclusion criteria included a positive skin prick test with a wheal diameter greater than 3 mm and a grass pollen-specific IgE level exceeding 0.70 kU/l; among these, 20 patients had undergone allergen immunotherapy (AIT) prior to their recruitment (Table 2S). Additionally, 27 non-allergic individuals with no clinical history of chronic rhinosinusitis were included as controls. The ethics commission of the Technical University of Munich approved the study (5534/12), and all participants provided written informed consent in accordance with the Helsinki Declaration.

PBMCs were isolated through density-gradient centrifugation and preserved through cryopreservation until the time of analysis. A structured timeline for sample collection was adhered to during the study. Blood samples were collected at baseline, just before, and six hours following the first and last top-dose injections during AIT's pre-season in the first year. Of note, this time point carries an increased number of allergen-specific cells due to the therapeutic injection of allergens (PMID: 12871638). Further samples were taken biannually during the maintenance phase, once during and once after the grass pollen season, over three subsequent years. Each sample collection point consisted of eight samples, but due to patient dropouts, the samples were not connected between different time points, rendering the analysis cross-sectional in nature.

**Sputum sample collection**

The study and the methodology have been previously described (35, 36). The sputum sample collected during grass pollen season were utilised in this study. All assessments took place at the Allergy Section of the Department of Otolaryngology at TUM School of Medicine in Munich, Germany. Data was collected from two non-interventional observational studies conducted over three years, both of which received approval from the Institutional Review Board (IRB) of TUM School of Medicine (numbers 5534/12 and 5156/11). Informed written consent was obtained from all participants. The study comprised 44 healthy individuals and 80 patients suffering from grass pollen-related allergic rhinitis (AR). Among these, 37 had AR without asthma (with 21 receiving AIT), whereas 43 had AR along with concurrent asthma (AA), of whom 22 were undergoing AIT. GINA scores were evaluated for the AA patients, and additional information can be found in Table 1S. Asthma diagnoses relied on prior evaluations by medical professionals and a history of symptoms such as shortness of breath, chest tightness, and coughing during pollen exposure, or a recorded positive response to bronchodilation tests. Patients identified with grass pollen allergies met ARIA criteria, presenting moderate to chronic persistent allergic rhinitis for more than two years during pollen season, a wheal greater than 3 mm, and grass pollen-specific IgE levels exceeding 0.70 kU/l. To limit the effects of medications, patients stopped using inhaled corticosteroids a week prior to sampling, although AIT treatment continued. All participants had good lung function (FEV1% > 70%), and those receiving AIT had been in therapy for at least one year. No severe or uncontrolled asthmatics were included into the study (FEV1 < 70%) Participants filled out the mini rhino conjunctivitis quality of life questionnaire (mRQLQ) and underwent pulmonary function tests along with sputum induction. Following an inhalation of salbutamol, subjects inhaled progressively increasing concentrations of nebulised hypertonic saline. The samples were resuspended in a sputolysin working solution (Merck Millipore) at a 4:1 volume-to-weight ratio of the sputum plug, then incubated on ice for 15 minutes, mixed with PBS (2:1 ratio), and filtered through a 70µm filter. The resulting filtrate was centrifuged at 790×g for 10 minutes without a brake to eliminate cells, which were then stored in RNA Cell Protect Reagent (Qiagen, Hilden, Germany) at -80°C for RNA analysis, while the supernatants were also preserved at -80°C. The quantity of extracted sputum varied among patients, which limited the types of analyses that could be performed and resulted in varying sample sizes across different tests.

**Flow Cytometry**

Flow cytometry was performed on murine bronchoalveolar lavage (BAL), lung, spleen, human sputum, and human PBMC samples as indicated in the Results section. Antibodies and staining panels are detailed in **Table 3S**.

Cells were first stained with a viability dye to exclude dead cells. Fc receptor blocking (where applicable) was performed using TruStain FcX (1:20; **BioLegend, San Diego, CA, USA**). Surface staining was carried out by incubating cells with fluorochrome-conjugated antibodies (typically 1:200 dilution) for 30 min at room temperature in FACS buffer (PBS supplemented with 2% FBS). For intracellular staining, surface-stained cells were fixed and permeabilized using Fix/Perm reagents, followed by staining with antibodies against intracellular proteins (cytokines diluted 1:100; transcription factors diluted 1:50) in PermWash buffer. Isotype controls were included to monitor non-specific binding.

Compensation was performed using antibody capture beads stained with individual fluorochromes. Data were acquired on either a BD LSRFortessa (BD Biosciences, Heidelberg, Germany) or a Cytek Aurora (Cytek Biosciences, Fremont, USA) and analyzed using FlowJo software (Ashland, OR, USA). Gating strategies are shown in Supplementary **Figures 1S, 3S, 6S, 9S** **and 12 S.**

**Sample-specific conditions:**

**Murine samples; Human sputum:** Cells were directly stained as described above using the antibodies described in Table 3S-A and analyzed on the BD LSRFortessa.

**Human PBMCs – PACIFIC cohort (anti-PD1 blockade):** Cells were cultured for 24 h in culture medium with plate-bound anti-CD3 (UCHT1, 5 µg/mL) and soluble anti-CD28 (CD28.2, 1 µg/mL) in the presence of either nivolumab (1 µg/mL, 5C4.B8, Absolute Antibody, UK) or mock IgG (4-4-20, Absolute Antibody, UK). Brefeldin A (BFA, 3 µg/mL) was added for the final 4 h of culture before staining and analysis on the BD LSRFortessa. The antibodies used are described in Table 3S-B and C.

**Human PBMCs – PACIFIC cohort (TNF profiling):** PBMCs were seeded in plates coated with anti-CD3 (1 µg/mL) and anti-CD28 (2 µg/mL) antibodies and incubated for 24h at 37°C. Brefeldin A (BFA, 3 µg/mL) was added for the final 4h of culture prior to staining. Cells were subsequently stained, fixed, permeabilized, and incubated with intracellular antibodies as described above. Samples were acquired on a Cytek Aurora spectral flow cytometer. The antibodies used are described in Table 3S-D.

**Human PBMCs – PACIFIC cohort (*ex vivo* LT-α stimulation):**

PBMCs isolated from participants of the PACIFIC cohort were stimulated *ex vivo* under two experimental conditions. For LT-α stimulation alone, PBMCs were seeded in plates coated with anti-CD3 (1 µg/mL) and anti-CD28 (2 µg/mL) antibodies and soluble lymphotoxin-α (LT-α; 1 µg/mL; Thermo Fisher Scientific) was added to the culture medium for 24h at 37°C. For TNFR2 blockade experiments, PBMCs were pre-incubated with anti-TNFR2 blocking antibody (5 µg/mL; Abcam, Cambridge, UK) for 1h at 37°C and subsequently transferred to anti-CD3 anti-CD28-coated plates. Soluble LT-α (1 µg/mL) was added to the culture medium, and cells were incubated for 24h. Brefeldin A (BFA, 3 µg/mL) was added during the final 4h of culture to all conditions. Cells were stained, fixed, permeabilized, and incubated with surface and intracellular antibodies as described above. Samples were acquired on a Cytek Aurora. The antibodies used are listed in Table 3S-D.

**Single-cell mRNA Sequencing**

Single-cell mRNA sequencing was performed on PBMCs derived from the PACIFIC cohort mentioned previously. The blood samples collected at baseline (T0) and after the AIT during the maintenance phase (T6) were utilised. PBMCs were stimulated *in vitro* using anti-CD3 (1µg/mL) and anti-CD28 (2µg/mL) coated plates for 24 hours prior to cell sorting with specific antibodies for human CD3, CD4 and CCR6 (BioLegend, San Diego, CA, USA; Supplementary Table 3) using BD FACSAria Fusion Flow Cytometer (BD Biosciences, Heidelberg, Germany). CD3+CD4+CCR6+ live cells were subsequently subjected to single-cell 5' transcriptome analysis. To this end, single-cell libraries were generated using the Chromium Next GEM Single Cell 5' v2 (Dual Index) kit (10x Genomics, Leiden, The Netherlands) along with the Chromium Controller for single-cell partitioning, adhering to the manufacturer's instructions. Quality control for the libraries was performed using the Bioanalyzer High Sensitivity DNA Analysis (Agilent, Waldbronn, Germany) and the KAPA Library Quantification Kit (Hoffmann-La Roche, Basel, Switzerland) to confirm appropriate fragment size and concentration prior to sequencing. Libraries that met these quality standards were sequenced on the NovaSeq 6000 platform (Illumina, San Diego, CA) at the Helmholtz Center Munich Sequencing Core Facility.

**Single-cell mRNA sequencing data processing and analysis**

Raw sequencing data were processed with the CellRanger toolkit (10x Genomics) to demultiplex and align the reads to the human reference genome (hg38). Unique Molecular Identifiers (UMIs) were quantified, and barcode artifacts were removed. A quality control step ensured that only cells expressing at least 200 genes were retained, and cells with a high mitochondrial gene content (>10%) were excluded to eliminate potentially stressed or dying cells. Genes expressed in fewer than 10 cells were also filtered out. The initial dataset comprised 60,731 cells from 7 samples (3 donors across 2 conditions each, with one donor lacking a condition). After the initial processing, we selected subsets of the main object based on the expression of key marker genes to isolate three populations of interest: Tr17 cells (FOXP3 > 0.6 & IL17A > 0.6), Th17 cells (IL17A > 0.6 & IL17F > 0.6 & FOXP3 < 0.6), and Tregs (FOXP3 > 0.6 & IL17A < 0.6 & IL17F < 0.6). These subsets were then merged into a new Seurat object containing 5,278 cells with 20,717 features from 4 samples. For this combined dataset, we applied data normalization using the LogNormalize method (Seurat v5.2.1) with a scale factor of 10,000. We regressed out ribosomal and mitochondrial gene content to mitigate technical variability. Highly variable genes were identified with the FindVariableFeatures function (selection.method = "vst", nfeatures = 2000) (Table 4S). Dimensionality reduction was executed via Principal Component Analysis (PCA), selecting the top 15 principal components for subsequent analysis. We corrected for batch effects using the RPCA integration method and visualized the integrated data utilizing Uniform UMAP. Cells were clustered with the Louvain algorithm at a resolution of 0.5, and marker genes were depicted through dot plots and feature plots to elucidate the gene expression profiles of each T cell subset.

**Pseudotime trajectory analysis**

Pseudotime trajectories were inferred using Monocle3 (v1.3.7). Cells were ordered along pseudotime based on entropy, and trajectories were visualised on UMAP and force-directed layouts. Root cells were identified as those with the highest entropy, and differentiation paths were reconstructed for conditions control and treatment. force-directed layouts were generated using ggplot2 (v3.5.1), patchwork (v1.3.0), and igraph (v2.1.4). Custom color schemes were applied to highlight specific subsets and pseudotime progression.

**Cell-cell communication analysis:**

Cell-cell communication networks were constructed and analysed using CellChat (v2.1.2) following the established pipeline for comparison analysis of multiple datasets (37). Separate CellChat objects were created for conditions control and AIT treated cells, using the human CellChatDB database with focus on secreted signalling molecules. After identifying overexpressed genes and interactions, communication probabilities were computed and filtered to retain significant interactions (min. 10 cells per group). The objects were then merged for comparative analysis. Differential cell-cell communication was evaluated through rankNet analysis, with circle plots and heatmaps visualising changes in interaction patterns between conditions. Specific signalling pathways (TNF and LT) were examined using chord diagrams and contribution analysis to identify key ligand-receptor pairs. Differential expression analysis was mapped onto communication networks to identify upregulated signalling.

**Sensitivity Analysis**

For sensitivity analysis, linear regression of logit-transformed Th17 frequencies was performed with age and sex as covariates. Predicted values were backtransformed to percentages for interpretability. Analyses were conducted in R version 4.5.1 (2025-06-13) using the packages tidyverse (v2.0.0), broom (v1.0.9), and performance (v0.15.1).”

**Statistics**

GraphPad Prism 10.4.1 (GraphPad Software, La Jolla, CA, USA) was used to perform the statistical analysis. Kruskal-Wallis test and two-tailed Mann-Whitney *U* test were used to evaluate statistical significance. Statistical significances were depicted as p-values *p<0.05, p<0.01, and *p<0.001.
