# Supplementary material for: TNF Pathway‐Mediated Tolerogenic T‐Cell Trajectory Driven by Allergen Immunotherapy
Source: Allergy. 2026 Apr 30;81(6):2184–97. doi: 10.1111/all.70367 (PMC13256266; doi:10.1111/all.70367)
Supplement: Supplementary file 2 — Table S1: Characteristics of allergic rhinitis and allergic asthmatic patients with without allergen immunotherapy from whom sputum was collected. Table S2: Characteristics of grass pollen‐allergic patients with immunotherapy (PACIFIC study). Table S3: Monoclonal antibodies used in flow cytometry analyses. [file ALL-81-2184-s001.docx]

**TNF pathway-mediated tolerogenic T cell trajectory driven by allergen immunotherapy**

**Authors:** Helen S. Charles^1,2^, M.Sc, Amr A. Gabr^1,2,3^, Ph.D., Shu-Hung Wang^1^, M.D. Ph.D., Ulrich M. Zissler ^1,2^, Ph.D., Sonja Heine^1^, Ph.D, Alexander Heldner^1^, Ph.D., Sebastian Kotz^4^, M.D., Lisa Pechtold^4^, M.D., Lynn S. zur Bonsen^4^, MD, Dimitrii Pogorelov^1^; Ph.D., Josephine Kau^4^, M.D., Mirjam Plaschke^4^, M.D., Miriam Hills^5^, Ph.D., Ferdinand Guerth^1^, M.Sc., Madlen Oelsner^1^, Caspar Ohnmacht^1^, Ph.D., Francesca Alessandrini^1^, Ph.D., Simon Blank^1^, Ph.D., Adam M. Chaker^1,4^, M.D., Carsten B. Schmidt-Weber^1,2^, Ph.D., Constanze A. Jakwerth^1,2,*^, Ph.D.

**Affiliations:**

^1^ Center of Allergy and Environment (ZAUM), School of Medicine and Health, Technical University of Munich, and Helmholtz Munich, Munich, Germany

^2^ Member of the German Center for Lung Research (DZL), Germany

^3^ Department of Physiology, Faculty of Veterinary Medicine, Cairo University, Giza 12211, Egypt

^4^ Department of Otorhinolaryngology, TUM School of Medicine and Health, Klinikum rechts der Isar, Technical University of Munich, Munich, Germany

^5^ Department of Dermatology and Allergy Biederstein, School of Medicine, Technical University of Munich, Munich, Germany

*** Corresponding author:**

PD. Dr. Constanze A. Jakwerth

Center of Allergy and Environment (ZAUM),

Technische Universität and Helmholtz Center Munich,

Biedersteiner Str. 29, 80802 München, Germany

[constanze.jakwerth@tum.de](mailto:constanze.jakwerth@tum.de)

Phone: +49 89 41403472

**Supplementary Tables**

**Table S1 Characteristics of allergic rhinitis and allergic asthmatic patients with without allergen immunotherapy from whom sputum was collected**

|  | Control (n=20) | AR w/o AIT (n=6) | AR with AIT (n=10) | AA w/o AIT (n=8) | AA with AIT (n=11) |  |
| --- | --- | --- | --- | --- | --- | --- |
|  |  |  |  |  |  |  |
| Age [years] | 21.9 ± 1.48 | 21.0 ± 1.10 | 25.7 ± 3.02 | 24.4 ± 6.07 | 32.3 ± 7.18 |  |
| Sex (m/f) | 8/12 | 3/3 | 4/6 | 4/4 | 9/2 |  |
| GINA score | n.d. | n.d. | n.d. | 1.17 ± 0.27 | 0.38 ± 0.16 |  |
| mRQLQ Score | 0.19 ± 0.38 | 2.27 ± 0.88 | 1.92 ± 1.13 | 2.61 ± 1.26 | 1.18 ± 0.86 |  |
| Total IgE [IU/L] | 42.18 ± 38.47 | 263.33 ± 292.56 | 115.40 ± 166.41 | 271.88 ± 251.86 | 304.64 ± 206.43 |  |
| PT/CAP to |  | | | | |  |
| Grass | 0 | 16/16 | 21/21 | 21/21 | 22/22 |  |
| Birch | 0 | 14/13 | 15/12 | 17/15 | 15/14 |  |
| HDM | 0 | 10/9 | 8/9 | 9/9 | 6/6 |  |
| Cat | 0 | 5/3 | 8/5 | 13/11 | 10/8 |  |
| FVC [L] | 4.55 ± 0.70 | 5.36 ± 0.89 | 4.91 ± 0.84 | 4.79 ± 1.01 | 5.13 ± 1.00 |  |
| FVC [%] | 97.85 ± 13.38 | 106.00 ± 13.12 | 106.60 ± 12.83 | 101.63 ± 11.91 | 107.91 ± 13.67 |  |
| FEV [L] | 3.87 ± 0.60 | 4.47 ± 0.79 | 4.09 ± 0.64 | 3.79 ± 0.56 | 4.06 ± 0.84 |  |
| FEV_1_ [%] | 96.85 ± 12.34 | 102.17 ± 11.59 | 102.10 ± 11.91 | 96.38 ± 13.45 | 104.36 ± 15.45 |  |
| FEV_1_/FVC ratio | 85.13 ± 6.35 | 83.45 ± 5.87 | 83.38 ± 5.21 | 79.44 ± 7.12 | 79.22 ± 6.98 |  |
| Sputum cells / ml (x10^4^) | 132.53 ± 65.94 | 251.33 ± 238.77 | 139.65 ± 71.34 | 154.69 ± 90.00 | 144.59 ± 65.94 |  |
| Macrophages (%) | 50.95 ± 2.12 | 51.33 ± 1.97 | 51.10 ± 2.05 | 50.88 ± 2.23 | 51.27 ± 1.89 |  |
| Lymphocytes (%) | 2.10 ± 0.45 | 2.00 ± 0.52 | 2.00 ± 0.47 | 2.13 ± 0.50 | 2.00 ± 0.44 |  |
| Neutrophils (%) | 46.85 ± 2.01 | 46.67 ± 2.12 | 46.90 ± 1.88 | 46.75 ± 2.34 | 46.91 ± 2.07 |  |
| Eosinophils (%) | 0.10 ± 0.05 | 0.00 ± 0.00 | 0.00 ± 0.00 | 0.25 ± 0.10 | 0.18 ± 0.08 |  |

Plus-minus values indicate SD

**Abbreviations:** AR, allergic rhinitis; AIT, allergen immunotherapy; AA, allergic asthma; GINA, Global Initiative for Asthma; n.d., not determined; mRQLQ, modified Rhinoconjunctivitis Quality of Life Questionnaire; IgE, immunoglobulin E; PT/CAP, prick test/serum specific IgE (CAP system); HDM, house dust mite; FVC, forced vital capacity; FEV1, forced expiratory volume in 1 second.

| **Table S2 Characteristics of grass pollen-allergic patients with immunotherapy (PACIFIC study)**   \| **Characteristic** \| **Immunotherapy Group n=32** \| **Non-Allergic Controls n=22** \| \| --- \| --- \| --- \| \|  \| \| **Age (years)*** \| 25.59 ± 6.26 \| 26.36 ± 5.07 \|  \| \| **Male sex (%)** \| 16 (50%) \| 9 (41 %) \|  \| \| **Skin Prick Test ( positive %)** \| \| \|  \| \| **Grass** \| 100% \| 0 % \|  \| \| **Birch** \| 63% \| 0% \|  \| \| **House Dust Mite** \| 28% \| 0% \|  \| \| **Total IgE** \| 147.1 ± 167.1 \| 18.63 ± 19.67 \|  \| \| **Grass Specific IgE** \| 32.74 ± 27.88 \| 0.00 ± 0.01 \|  \| \| **Birch Specific IgE** \| 8.75 ± 15.9 \| 0.15 ± 0.16 \|  \| \| **House Dust Mite Specific IgE** \| 2.85 ± 10.1 \| 0.10 ± 0.19 \|  \| \| **Allergic Asthma (%)** \| 14 (44%) \| 0% \|  \| |
| --- | --- | --- | --- | --- | --- | --- | --- | --- | --- | --- | --- | --- | --- | --- | --- | --- | --- | --- | --- | --- | --- | --- | --- | --- | --- | --- | --- | --- | --- | --- | --- | --- | --- | --- | --- | --- | --- | --- | --- | --- | --- | --- | --- | --- | --- | --- | --- | --- |
| Plus-minus values indicate SD  *at informed consent procedure and inclusion into study |

**Table S3 Monoclonal antibodies used in flow cytometry analyses**

1. **Fluorochrome labelled anti-mouse antibodies**

| Specificity | Fluorochrome | Clone | Manufacturer |
| --- | --- | --- | --- |
| CD279 (PD-1) | PE-Cy7 | J43 | Thermo Fisher Scientific, Waltham, MA, USA |
| CD152 (CTLA-4) | PE-eFluor610 | UC10-4B9 | Thermo Fisher Scientific, Waltham, MA, USA |
| Ki67 | PE-Cy7 | B56 | BD Biosciences, Franklin Lakes, NJ, USA |
| CD196 (CCR6) | BV421 | 29-2L17 | BioLegend, San Diego, CA, USA |
| Foxp3 | PerCP-Cy5.5 | FJK-16s | Thermo Fisher Scientific, Waltham, MA, USA |
| RORgt | PE | AFKJS-9 | Thermo Fisher Scientific, Waltham, MA, USA |
| IL-17A | BV605 | TC11-18H10 | BD Biosciences, Franklin Lakes, NJ, USA |
| TCF-1 | PE | S33-966 | BD Biosciences, Franklin Lakes, NJ, USA |
| CD3 | FITC | 145-2C11 | BD Biosciences, Franklin Lakes, NJ, USA |
| CD4 | AF700 | RM4-5 | BD Biosciences, Franklin Lakes, NJ, USA |
| CD45 | APC-eFluor780 | 30-F11 | Thermo Fisher Scientific, Waltham, MA, USA |
| CD44 | BV605 | IM7 | BioLegend, San Diego, CA, USA |

1. **Unconjugated antibodies**

| Specificity | Clone | Manufacturer |
| --- | --- | --- |
| α-human CD3 | UCHT1 | BD Biosciences, Franklin Lakes, NJ, USA |
| α-human CD28 | CD28.2 | BD Biosciences, Franklin Lakes, NJ, USA |
| α-human PD-1 (Nivolumab) | 5C4.B8 | Absolute Antibody, Wilton, UK |
| α-Fluorescein, human IgG4-S228P | 4-4-20 | Absolute Antibody, Wilton, UK |
| α-human TNFR | MR2-1 | Abcam, Cambridge, UK |

1. **Fluorochrome labelled anti-human antibodies (Human Sputum and a-PD-1 Blockade assay)**

| Specificity | Flurochrome | Clone | Manufacturer |
| --- | --- | --- | --- |
| CD3 | APC-Cy7 | HIT3a | BioLegend, San Diego, CA, USA |
| CD4 | BV421 | RPA-T4 | BioLegend, San Diego, CA, USA |
| CD45RA | PerCP-Cy5.5 | HI100 | BioLegend, San Diego, CA, USA |
| PD-1 | BV650 | EH12.2H7 | BioLegend, San Diego, CA, USA |
| CTLA-4 | PE-Cy7 | L3D10 | BioLegend, San Diego, CA, USA |
| TCF-1 | PE | S33-966 | BD Biosciences, Franklin Lakes, NJ, USA |
| IL-17A | BV711 | BL168 | BioLegend, San Diego, CA, USA |
| FOXP3 | PerCP-Cy5.5 | PCH101 | Thermo Fisher Scientific, Waltham, MA, USA |

1. **Fluorochrome labelled anti-human antibodies (TNF/LT Profiling)**

| Specificity | Flurochrome | Clone | Manufacturer |
| --- | --- | --- | --- |
| CD120b (TNFR1b) | PE | 3G7A02 | BioLegend, San Diego, CA, USA |
| CD127 (IL-7Ra) | BV785 | A019D5 | BioLegend, San Diego, CA, USA |
| CD152 (CTLA-4) | PE-Cy7 | L3D10 | BioLegend, San Diego, CA, USA |
| CD196 (CCR6) | BV711 | G034E3 | BioLegend, San Diego, CA, USA |
| CD25 | BV650 | BC96 | BioLegend, San Diego, CA, USA |
| CD270 | FITC | 004 | Miltenyi Biotec, Bergisch Gladbach, Germany |
| CD279 (PD-1) | BV605 | EH12.2H7 | BioLegend, San Diego, CA, USA |
| CD3 | APC-Cy7 | HIT3a | BioLegend, San Diego, CA, USA |
| CD4 | PE Dazzle 594 | RPA-T4 | BioLegend, San Diego, CA, USA |
| CD45RA | BUV395 | HI100 | Thermo Fisher Scientific, Waltham, MA, USA |
| FOXP3 | Per CP Cy5.5 | PCH101 | Thermo Fisher Scientific, Waltham, MA, USA |
| IL-17A | PE-Cy5 | BL168 | BioLegend, San Diego, CA, USA |
| IL-2 | BV421 | MQ1-17H12 | BioLegend, San Diego, CA, USA |
| Ki-67 | eFluor 506 | PCH101 | Thermo Fisher Scientific, Waltham, MA, USA |
| TNF-Beta | APC | 359-81-11 | Thermo Fisher Scientific, Waltham, MA, USA |

**Table S4** can be found as separate table file.
