# Supplementary material for: TNF Pathway‐Mediated Tolerogenic T‐Cell Trajectory Driven by Allergen Immunotherapy
Source: Allergy. 2026 Apr 30;81(6):2184–97. doi: 10.1111/all.70367 (PMC13256266; doi:10.1111/all.70367)
Supplement: Supplementary file 4 — Figure S1: Representative sequential gating strategy of OVA Mice model with allergic airway inflammation (AAI) and AAI treated with allergen immunotherapy (AIT). Figure S2: PD‐1 and CTLA‐4 Expression in Th17, Tr17 and Treg Populations in the Spleen. Figure S3: Representative sequential gating strategy of sputum samples of allergic rhinitis and allergic asthmatic patients with and without AIT. Figure S4: Effect of AIT on Th17, Tr17 and Treg populations in the sputum of Asthmatic patients. Figure S5: Modulation of Th17 Cell Phenotype in Blood Following AIT. Figure S6: Representative sequential gating strategy on the PBMCs isolated from allergic patients through the course of AIT. Figure S7: Role of AIT on the stemness of exhausted Th17 Cell Phenotype in blood. Figure S8: Notable alterations in receptors‐ligands‐mediated communications between different cell types before and after AIT. Figure S9: Representative sequential gating strategy on the PBMCs isolated from grass‐pollen allergic patients before and after 1 year of AIT. Figure S10:. Checkpoint and proliferation markers are largely unchanged after 1 year of AIT. Figure S11:. TNFR‐LT‐α marker expression in iTregs after 1 year of AIT. Figure S12: Representative staining controls for PACIFIC Cohort TNF Pathway Profiling flow cytometry panel involving appropriate FMO and Single stain layover for individual markers. Figure S13:. Subset‐dependent changes in TNF signalling following LT‐α stimulation after 1 year of AIT. Figure S14: TNFR2 blockade attenuates LT‐α‐induced signalling and checkpoint remodelling across T‐cell subsets. [file ALL-81-2184-s003.docx]

**TNF pathway-mediated tolerogenic T cell trajectory driven by allergen immunotherapy**

**Authors:** Helen S. Charles^1,2^, M.Sc, Amr A. Gabr^1,2,3^, Ph.D., Shu-Hung Wang^1^, M.D. Ph.D., Ulrich M. Zissler ^1,2^, Ph.D., Sonja Heine^1^, Ph.D, Alexander Heldner^1^, Ph.D., Sebastian Kotz^4^, M.D., Lisa Pechtold^4^, M.D., Lynn S. zur Bonsen^4^, MD, Dimitrii Pogorelov^1^; Ph.D., Josephine Kau^4^, M.D., Mirjam Plaschke^4^, M.D., Miriam Hills^5^, Ph.D., Ferdinand Guerth^1^, M.Sc., Madlen Oelsner^1^, Caspar Ohnmacht^1^, Ph.D., Francesca Alessandrini^1^, Ph.D., Simon Blank^1^, Ph.D., Adam M. Chaker^1,4^, M.D., Carsten B. Schmidt-Weber^1,2^, Ph.D., Constanze A. Jakwerth^1,2,*^, Ph.D.

**Affiliations:**

^1^ Center of Allergy and Environment (ZAUM), School of Medicine and Health, Technical University of Munich, and Helmholtz Munich, Munich, Germany

^2^ Member of the German Center for Lung Research (DZL), Germany

^3^ Department of Physiology, Faculty of Veterinary Medicine, Cairo University, Giza 12211, Egypt

^4^ Department of Otorhinolaryngology, TUM School of Medicine and Health, Klinikum rechts der Isar, Technical University of Munich, Munich, Germany

^5^ Department of Dermatology and Allergy Biederstein, School of Medicine, Technical University of Munich, Munich, Germany

*** Corresponding author:**

PD. Dr. Constanze A. Jakwerth

Center of Allergy and Environment (ZAUM),

Technische Universität and Helmholtz Center Munich,

Biedersteiner Str. 29, 80802 München, Germany

[constanze.jakwerth@tum.de](mailto:constanze.jakwerth@tum.de)

Phone: +49 89 41403472

**Supplementary Figures and Figure Legends**

**Figure S1** *Representative sequential gating strategy of OVA Mice model with allergic airway inflammation (AAI) and AAI treated with allergen immunotherapy (AIT).*

Flow cytometry plots illustrate the gating strategy used to identify T cell subsets from lung tissue. Singlets and lymphocytes were first gated based on forward and side scatter characteristics. Viable lymphocytes were selected using a viability dye exclusion method, followed by gating on CD45+ leukocytes. Within this population, CD3+ CD4+ T helper cells were further defined. Subsequent gating revealed FoxP3+ regulatory T cells (Tregs), RORγt+ Th17-like cells, and dual-expressing FoxP3+RORγt+ Tr17 cells.

**Figure S2** *PD-1 and CTLA-4 Expression in Th17, Tr17, and Treg Populations in the Spleen*.

Flow cytometric analysis of PD-1⁺ and CTLA-4⁺ Th17, Tr17, and Treg cell populations in the spleen (Control n=8; AAI n=8; AAI+AIT n=8). PD-1 expression: Quantification of PD-1⁺ Th17 (A), Tr17 (B), and Treg (C) cells. CTLA-4 expression: Quantification of CTLA-4⁺ Th17 (D), Tr17 (E), and Treg (F) cells.

**Figure S3** *Representative sequential gating strategy of sputum samples of allergic rhinitis and allergic asthmatic patients with and without AIT*.

Initially, singlets and lymphocytes were identified based on forward and side scatter, followed by the selection of viable lymphocytes using a viability dye exclusion method. Within the live cell population, CD3+ T cells were isolated, and the CD4+ T helper (Th) subset was subsequently defined. Representative flow cytometry plots then illustrate the identification of FoxP3+ regulatory T cells (Tregs) within the CD4+ subset, as well as the delineation of IL-17+ Th17 and Tr17 subsets.

**Figure S4** *Effect of AIT on Th17, Tr17, and Treg populations in the sputum of Asthmatic patients.* Representative flow cytometry-based quantification of Th17 (Fig. 3S A), Tr17 (Fig. 3S B), Treg (Fig. 3S C) populations in the sputum sample of healthy control (n=20) and asthmatic patients before (n=8) and after AIT (n=11). Statistical analysis was performed using Mann-Whitney U test and the statistical significance is indicated by * p< 0.05, ** p< 0.01, *** p< 0.001.

**Figure S5** *Modulation of Th17 Cell Phenotype in Blood Following AIT*

(A) Representative flow cytometry plots showing IL-17A, IL-2, PD-1, and CTLA-4 expression in Th17 cells from baseline and over the course of AIT :T0 to T9 (T0 n=8; T1 n=6; T3 n=7; T4 n=6; T5 n=9; T6 n=9; T7 n=9; T8 n=9; T9 n=8). (B) Quantification of IL-17A+ Th17 cells over the course of AIT, including the top dose phase and follow-up years. (C-F) Frequency of PD-1+ Th17 cells (C), IL-2+ PD-1+ Th17 cells (D), CTLA-4+ Th17 cells (E), and IL-2+ CTLA-4+ Th17 cells (F) at different time points during the course of AIT. (G-H) IL-2 expression in Th17 cells (G) and IL-2+ PD-1+ Th17 cells (H) following an in-vitro anti-PD-1 blockade, comparing mock IgG and Nivolumab-treated conditions. Blue shading indicates the top-dose phase of AIT, while yellow shading marks the follow-up years. Statistical analysis was performed using Mann-Whitney U test and Kruskal-Wallis test and the statistical significance is indicated by * p< 0.05, ** p< 0.01, *** p< 0.001. The data were not pooled (No. of Experiments = 1)

**Figure S6** *Representative sequential gating strategy on the PBMCs isolated from allergic patients through the course of AIT*.

Singlets were initially identified based on forward and side scatter (FSC-A vs. SSC), followed by gating for lymphocytes using FSC-A and FSC-H parameters. Viability dye exclusion was then used to select live cells, allowing the identification of CD3+ T cells within the viable lymphocyte population. CD4+ T helper (Th) cells were subsequently defined, and further gating was performed to distinguish Th17 cells based on IL-17A expression. Effector and memory subsets were assessed using CD45RA, while co-inhibitory receptors such as PD-1 and CTLA-4, as well as markers of stemness like TCF1 and KLRG1, were analyzed within the CD4+ population.

**Figure S7** *Role of AIT on the stemness of exhausted Th17 Cell Phenotype in blood.*

Frequency of TCF^+^ PD-1^+^ Th17 cells (A) TCF^+^ CTLA-4^+^ Th17 cells (B) and PD-1^+^ CTLA-4^+^ Th17 cells at different time points during the course of AIT. Statistical analysis was performed using Mann-Whitney U test and Kruskal-Wallis test and the statistical significance is indicated by * p< 0.05, ** p< 0.01, *** p< 0.001.

**Figure S8** *Notable alterations in receptors-ligands-mediated communications between different cell types before and after AIT.*

Heatmaps for selected signals contributing the most to incoming signalling patterns of different cell populations before AIT at T0 (A) and after AIT at T6 (B). Heatmaps for selected signals contributing the most to outgoing signalling patterns of different cell populations before AIT at T0 (C) and after AIT at T6 (D). Chord diagram showing all the significant interactions (Lignad-Receptor pairs) between different signalling pathways in Tr17 cells. The incoming communication pattern of the signalling pathways of Tr17 cells before AIT at T0 (E) and after AIT at T6 (F). The outgoing communication pattern of the signalling pathways of Tr17 cells before AIT at T0 (G) and after AIT at T6 (H). Violin Plot comparisons of the significant ligand receptors of the LT pathway before (red) and after (blue) AIT, which contribute to the signalling between the populations of interest: Th17, iTregs, iTregs (TR), and Tr17 subpopulations (I). Heatmap shows the relative importance of each cell type as sender, receiver, mediator and influencer in the TNF Pathway based on the computed four network centrality measures of different signaling pathways (J and L). Chord diagram for visualizing cellcell communication through LT signaling pathway before AIT at T0 (K) and after AIT at T6 (M). The lines represent changes in ligand-receptor interaction strengths and the color bars in the inner circles indicates targeting cell types of the outgoing signaling while noncolor part for incoming signaling.

**Figure S9** *Representative sequential gating strategy on the PBMCs isolated from grass-pollen allergic patients before and after one year of AIT*.

Sequential gating strategy applied to PBMCs isolated from grass-pollen allergic patients followed by *in vitro* stimulation with anti-CD3 and anti-CD28. Singlets were first identified using forward and side scatter (FSC-A vs. SSC-A), followed by gating for lymphocytes via FSC-A and FSC-H parameters. Viable cells were selected using a live/dead exclusion dye, and CD3⁺CD4⁺ T cells were subsequently defined. Within this population, FOXP3⁺ regulatory T cells (Tregs), IL-17A⁺ Th17 cells, and IL-17A⁺FOXP3⁺ Tr17 cells were identified. The contour plots shown are representative for the Treg population, including expression of TNFR2, CTLA-4, IL-2, LT-α, HVEM, and Ki-67.

**Figure S10.** *Checkpoint and proliferation markers are largely unchanged after one year of AIT.* Representative plots and quantification of HVEM,TNFR2, PD-1, CTLA-4, IL-2 and Ki-67 expression across Th17, Tr17 and Treg populations comparing baseline (T0) and one year post-AIT (T6) (n=28) (A-R). Statistical analysis was performed using Mann-Whitney U test and the statistical significance is indicated by * p< 0.05, ** p< 0.01, *** p< 0.001. Data represent a single experiment.

**Figure S11.** *TNFR-LT-α marker expression in iTregs after one year of AIT.*

Representative plots and quantification of iTregs and the different TNFR family markers comparing baseline (T0) and one year post-AIT (T6) (n=28) (A-F). Statistical analysis was performed using Mann-Whitney U test and the statistical significance is indicated by * p< 0.05, ** p< 0.01, *** p< 0.001. Data represent a single experiment.

**Figure S12** *Representative staining controls for PACIFIC Cohort TNF Pathway Profiling flow cytometry panel involving appropriate FMO and Single stain layover for individual markers*

**Figure S13.** *Subset-dependent changes in TNF signalling following LT-α stimulation after one year of AIT.* Flow cytometric quantification of HVEM⁺LT-α⁺ (A-C), TNFR2⁺LT-α⁺ (D-F), PD-1 (G-I), CTLA-4 (J-L), IL-2 (M-O), and Ki-67 (P-R) expression within Th17, Tr17, and Treg populations from grass pollen-allergic patients at baseline (T0) and after one year of allergen immunotherapy (T6) (n = 15). PBMCs were stimulated with anti-CD3/anti-CD28 alone or anti-CD3/anti-CD28 + LT-α. In each subfigure, the left panels correspond to baseline samples (T0) and the right panels to samples obtained after one year of AIT (T6). Each dot represents one patient. Statistical comparisons were performed using the two-tailed Mann-Whitney U test; significance is indicated as *p < 0.05, **p < 0.01, ***p < 0.001. Data are shown as mean ± SEM.

**Figure S14.** *TNFR2 blockade attenuates LT-α-induced signalling and checkpoint remodelling across T-cell subsets.* Flow cytometric quantification of HVEM⁺LT-α⁺ (A-C), TNFR2⁺LT-α⁺ (D-F), PD-1 (G-I), CTLA-4 (J-L), IL-2 (M-O), and Ki-67 (P-R) expression within Th17, Tr17, and Treg populations from grass pollen-allergic patients at baseline (T0) and after one year of allergen immunotherapy (T6) (n = 15). PBMCs were stimulated with anti-CD3/anti-CD28 + LT-α in the presence or absence of a TNFR2-blocking antibody. In each subfigure, the left panels correspond to baseline samples (T0) and the right panels to samples obtained after one year of AIT (T6). Each dot represents one patient. Statistical comparisons were performed using the two-tailed Mann-Whitney U test; significance is indicated as *p < 0.05, **p < 0.01, ***p < 0.001. Data are shown as mean ± SEM.
